# Dr. William Henry Bullis

**Published:** 1917-08

**Authors:** 


**Dr. William Henry Bullis,** Queen's University of Kingston, Ont., 1884, died at his home in Rochester, June 28, aged 59, following a surgical operation.

